# Strain-specific predation of *Bdellovibrio bacteriovorus* on *Pseudomonas aeruginosa* with a higher range for cystic fibrosis than for bacteremia isolates

**DOI:** 10.1038/s41598-022-14378-5

**Published:** 2022-06-22

**Authors:** Claudia Saralegui, Cristina Herencias, Ana Verónica Halperin, Juan de Dios-Caballero, Blanca Pérez-Viso, Sergio Salgado, Val F. Lanza, Rafael Cantón, Fernando Baquero, M. Auxiliadora Prieto, Rosa del Campo

**Affiliations:** 1 Department of Microbiology, Hospital Universitario Ramón Y Cajal, Instituto Ramón Y Cajal de Investigación Sanitaria (IRYCIS), CIBERINFEC, Madrid, Spain; 2 Microbial and Plant Biotechnology Department, Biological Research Center-Margarita Salas, CSIC, Ramiro de Maeztu 9, 28040 Madrid, Spain; 3 CIBERESP, Madrid, Spain

**Keywords:** Clinical microbiology, Microbial ecology

## Abstract

This work aimed to evaluate the predatory activity of *Bdellovibrio bacteriovorus* 109J on clinical isolates of *Pseudomonas aeruginosa* selected from well-characterized collections of cystic fibrosis (CF) lung colonization (n = 30) and bloodstream infections (BSI) (n = 48) including strains selected by genetic lineage (frequent and rare sequence types), antibiotic resistance phenotype (susceptible and multidrug-resistant isolates), and colony phenotype (mucoid and non-mucoid isolates). The intraspecies predation range (I-PR) was defined as the proportion of susceptible strains within the entire collection. In contrast, the predation efficiency (PE) is the ratio of viable prey cells remaining after predation compared to the initial inoculum. I-PR was significantly higher for CF (67%) than for BSI *P. aeruginosa* isolates (35%) probably related to an environmental origin of CF strains whereas invasive strains are more adapted to humans. I-PR correlation with bacterial features such as mucoid morphotype, genetic background, or antibiotic susceptibility profile was not detected. To test the possibility of increasing I-PR of BSI isolates, a polyhydroxyalkanoate depolymerase deficient *B.* *bacteriovorus* bd2637 mutant was used. Global median I-PR and PE values remained constant for both predators, but 31.2% of 109J-resistant isolates were susceptible to the mutant, and 22.9% of 109J-susceptible isolates showed resistance to predation by the mutant, pointing to a predator–prey specificity process. The potential use of predators in the clinical setting should be based on the determination of the I-PR for each species, and the PE of each particular target strain.

## Introduction

The “golden age of antibiotics” in the mid-twentieth century was followed by the emergence of pathogens resistant to almost all available antibiotics, leading to the current global crisis of multidrug-resistant (MDR) bacteria^1, 2^. Identifying alternative interventions to antimicrobials requires a complete understanding of the ecological rules governing the commensal microbiota, which includes natural competitors and predators^3–6^. In nature, predatory bacteria play an important role in maintaining population sizes by linking the production and removal of biomass in microbial communities, which in turn promotes the diversity of microorganisms and contributes to the global stabilization of the ecosystem^7, 8^. The ecological role of predators could also be exploited in the fight against clinical pathogens since they are dynamic microorganisms that undergo (as do their opponents) continuous physical, morphological, and metabolic adaptation to the ecosystem. This evolutionary reciprocity is the basis of coevolution, in which the adaptation of a player not only promotes change in its opponent, but the opponent's adaptation also generates selection as an evolutionary response to the first player^9^.

The successful development of predatory bacteria as “living antimicrobial agents” and a complete understanding of the predation mechanism depend on the characterization of their predation preferences, mainly their predation range and efficiency among different species. However, within a bacterial species, predation appears to be species-specific but not universal for all its lineages, which can be referred to as intraspecific predation range (I-PR). Again, within each strain or clone, the predator may have different predation efficient (PE), depending on the proportion of predated cells. This polymorphic predation behavior is fundamentally based on the composition of the species or individual prey cell envelope, eventually influenced by environmental conditions^10–12^. The identification of the factors driving prey preference and differences in predatory activity between and within species has so far remained limited, particularly in non-environmental bacterial collections^13, 14^.

The most studied bacterial predators are *Bdellovibrio* and -like organisms (BALOs), which are small vibrioid gram-negative aerobic bacteria, recently reclassified to the class of *Oligoflexia*, which belongs to the *Pseudomonadota* phylum^15^. Although BALOs were first isolated from soil, they are ubiquitous in nature and can be found in aquatic and terrestrial environments, including hypersaline systems^16^, biofilms^17^, mammalian intestines^18–20^, and, as we have shown, the lungs of cystic fibrosis (CF) patients^21^. In addition to the genetic detection of BALOs, several authors have documented the in vivo phenomenon of predation in human microbial ecosystems^22–25^.

*Pseudomonas aeruginosa* is the most studied prey of BALOs, and both predator and prey frequently share a common environmental origin. This genus is also one of the most significant human pathogens, included in the ESKAPE category (including *Enterococcus faecium*, *Staphylococcus aureus*, *Klebsiella pneumoniae*, *Acinetobacter baumannii*, *Pseudomonas aeruginosa,* and *Enterobacter *spp.) by its high prevalence in severe invasive infections, such as bacteremia, and their ability to acquire antibiotic multiresistance^26^. *P. aeruginosa* is also the most relevant and abundant microorganism involved in CF pathogenic lung colonization; particularly the so-called high-risk clones with multiresistance to antibiotics and those with mucoid morphotype due to alginate hyperproduction. The genetic and molecular characterization of *P. aeruginosa* isolates from CF^27^ and bacteremia^28^ origins have been previously addressed by our group, showing that CF isolates are more related to an environmental origin^29^.

Herein, we determined the I-PR of the reference strain *B. bacteriovorus* 109J against a comprehensive collection of 78 *P. aeruginosa* clinical isolates, of diverse patient origins, genetic backgrounds, colonial, and antibiotic-resistant phenotypes. We also explore the possibility of increasing I-PR on the BSI collection by modifying the predator genome, presenting as proof of concept a single-gene *B*. *bacteriovorus* mutant^30^, the strain bd2637 which harbors a deficient polyhydroxyalkanoate depolymerase (PHA) which was able to prey on some 109J -resistant strains, but in contrast, other 109J -sensitive strains showed resistance to predation by the mutant. We discuss possible mechanisms that might explain this effect, opening the door to genetic engineering of predators to overcome reduced predator susceptibility in *P. aeruginosa*.

## Results

### Prey’s clinical source influences predation activity by *B. bacteriovorus* 109J

**Figure 1:**
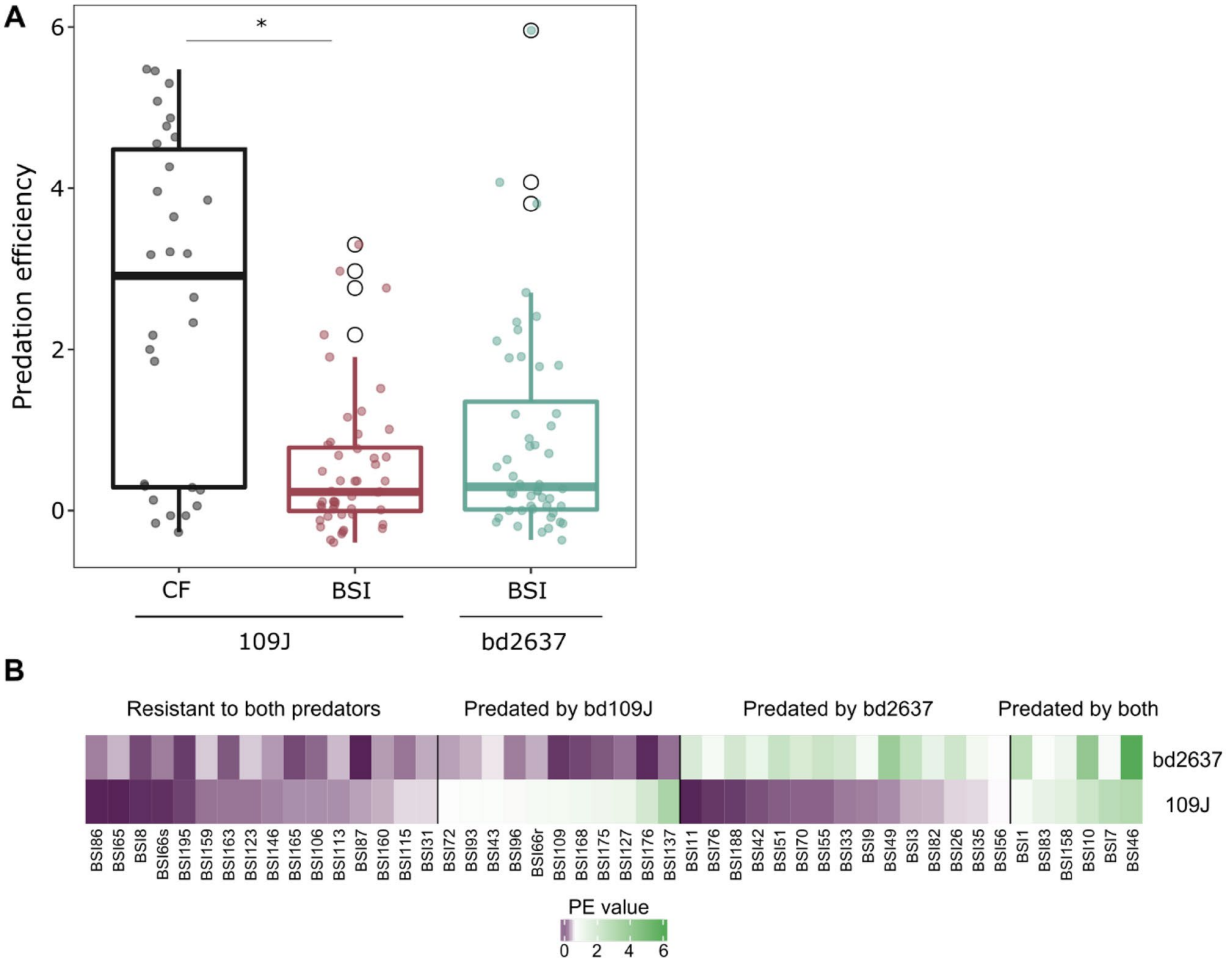
Predation susceptibility of *P. aeruginosa* collections. (**A**) Predation efficiency (PE) of *B. bacteriovorus* 109J on CF and BSI collection (*represents significance *p* < 0.0001) and PE of mutant strain bd2637 upon BSI collection (*p* > 0.05). (**B**) PE profile of the wildtype *B. bacteriovorus* 109J and mutant strain bd2637 upon BSI collection. Positive predation was considered when PE > 0.5. Each strain has a specific predation profile. White circles in BSI collection represent outliers.

A significantly higher I-PR value was observed in CF (20/30, 67.0%, Fig. S1) than in BSI isolates (17/48, 35.4%, *p* = 0.02, Fig. S2). In addition, median PE was higher for CF (median PE 3.91) than for BSI strains (median PE 1.10) (Mann–Whitney U test *p* < 0.0001) (Fig. 1A, Tables S1, and S2). The association of I-PR and the origin of the collection was also performed using linear model (lm, *p* < 0.0001). Correlation between predation activity and *P. aeruginosa* genetic lineage (Sequence Type; ST, obtained by MLST) was not observed (*p* > 0.05), with discrepancies in 6 of the 12 STs grouping more than one isolate from both collections [ST175 (1 susceptible prey out of a total of 3), ST253 (2/3), ST274 (1/2), ST532 (1/2), ST646 (2/3), and ST1017 (1/2)] (Tables S1 and S2). There was also no correlation with the mucoid (4/7 mucoid isolates) or non-mucoid phenotype of CF isolates (16/23 mucoid isolates) (*p* > 0.05) or with the antibiotic susceptibility phenotype (*p* > 0.05) (Fig. S3A and Table S2).

### Influence of the predator genetic variation

The predation ability of the *B.* *bacteriovorus* 109J wildtype strain was compared to those of the single-gene mutant bd2637 in the 48 BSI isolates, the less susceptible collection. There were no significant differences between I-PR values of mutant and wildtype strains (21/48, 43.8% vs. 17/48, 35.4%, *p* > 0.05); nor in the global median PE values (1.10 for *B. bacteriovorus* 109J *vs.* 1.8 for bd2637 mutant, *p* > 0.05) (Fig. 1A, Fig. S4, and Table S4). Interestingly, the range between both predators within the BSI collection differed: 16 isolates (33.3%) were resistant and 6 (12.5%) susceptible to both predators, whereas 15 (31.2%) were susceptible only to the bd2637 mutant, and finally 11 isolates (22.9%) were susceptible only to the wildtype predator (Fig. 1B).

There was no correlation between predation of bd2637 mutant and antibiotic susceptibility or any other prey feature (*p* > 0.05) (Fig. S3B and Table S3). These results were consistently observed in the replicates of each isolate and were not associated with any detected particular prey characteristic. Thus, only the deletion of the catalytic activity from the *bd2637* gene and the associated effects were responsible for the changes in prey susceptibility.

## Discussion

The use of BALOs as biological control agents in environmental and medical microbiological settings^18, 31^ has been suggested based on their lack of interaction with human cells^32–34^. As occurs with antibiotics, testing the individual in vitro susceptibility for prey and predator pairs of strains is a requirement, mainly when predation could be substantially affected by environmental or biological conditions, as we postulate herein. Predators have been studied primarily under controlled conditions (medium, pH, and temperature), and the knowledge on predation susceptibility of clinical isolates remains very limited. We focused our research on *P. aeruginosa* as this species was reported to have a limited ability to serve as a prey for *B.* *bacteriovorus*^35^, and therefore we expected a higher diversity in predator–prey response. In addition, *P. aeruginosa,* ubiquitous in nature (where most *Bdellovibrio-Pseudomonas* interactions are expected to occur), is a significant human pathogen frequently harboring mechanisms of antibiotic resistance. Also, previous studies of our group facilitated the building-up of a comprehensive and well-characterized collections including both frequent and infrequent lineages from CF^27^ and BSI^28^, accordingly to their ST. Note that the same *P. aeruginosa* ST can be found in different habitats, such as environmental and nosocomial niches (human microbiota of patients, built environments), but some lineages seem to be highly adapted to particular clinical environments. This is the case of chronic colonization of CF-patients airways, where the associated lineages are close to strains of environmental origin, as we have previously shown^29^. We did not find a correlation between ST (sequence types) and I-PR.

A notable result of our study is the significantly higher qualitative (I-PR, proportion of predated strains in the collections) and quantitative (PE, proportion of cells predated per individual strain) susceptibility of CF isolates in comparison with BSI ones. In addition, there was no correlation between predation and the colonial morphology, mucoid vs. non-mucoid, according to previous reports^36^, or antibiotic susceptibility patterns, or even genetic ST lineages. We cannot exclude that predation differences in the same ST lineage might derive from other genetic features such as the presence of pathogenicity islands or prophages.

The existence of highly specialized predators in human microbiota cannot be ruled out since all available data have been obtained from environmental predators. Human microbial ecosystems, however, probably have the same rules of population control based on predation. Predators with a human origin could be more suitable for limiting the well-adapted human pathogens^21^, despite that in a complex and diverse ecosystem, the preference for particular prey would be a dynamic feature^37^. Although experiments are often conducted using individual lineages, the use of mixed populations should be a future goal to validate prey specificity in a community and the consequences on microbial population structure. This work indicates that quantitative predation, determined as relative PEs might differ among members of an (apparently) homogeneous population and could be critical to understand the dynamics of bacterial ensembles composed by different prey and predators.

Only 8 *B.* *bacteriovorus* genomes have been entered into public databases, all of them from an environmental origin (soil, rivers, seawater, wastewater), but the contribution of the predators’ genetic background in the PE is a pending issue. To elucidate that, we used the mutant bd2637, to test the possibility of engineering more efficient, wider I-PR and PE predators^30^ on BSI isolates. We show that this mutant has a different spectrum than the wildtype strain, but how might that occur? The genotype of the bd2637 strain corresponds to a single deletion of the *bd2637* gene, which encodes a putative PHA depolymerase responsible for the degradation of biopolymers. Hypothetically, this mutant should have less access to the rich nutritional resources of polyhydroxyalkanoate (PHA) and consequently less fitness. However, the reality is probably more complex. Inside the “common metabolism” when the predator is placed into the prey, massive PHA degradation yields an ATP excess, which is known to increase reactive oxygen species, which can oxidize and damage DNA, carbohydrates, proteins, or lipids^38, 39^. Excess ATP also triggers respiration, but in small cells (such as *Bdellovibrio*) the question is whether high ATP levels are incompatible with the maintenance of respiration if the surface of the cell cannot allocate enough respiratory proteins^40, 41^. In short, PHA defective mutants might have unexpected metabolic consequences^42–46^ influencing predation activity, and research in this field could help to identify and eventually overcome predation resistance factors in *P. aeruginosa*. It is worth noting that the prey range (intra- or interspecies range) does not depend only on the prey susceptibility but also on the predator specificity, which highlights the importance of specific predator–prey susceptibility testing, as their interaction and co-evolution may overcome predation and resistance, respectively^47, 48^. A promising perspective for future work is trying to see if predation host-specificity and predation rate might differ when predators are propagated in bacterial species/clones isolated from closely related or different niches. The evolution of the predator in coevolving bacterial communities might result in an accelerated genomic predator and host evolution^49^.

The prey or predator determinants responsible for predation specificity have not been completely elucidated. However, the hydrolytic arsenal of *B. bacteriovorus* plays a crucial role in predation activity, given that it determines the success of their lifecycle^30, 50^. Characterization of each predator’s specific prey spectrum is a requirement for the clinical use of predators as “living antimicrobial agents”. Studies have reported that different BALOs lineages and predators isolated from different niches have different prey spectra^51, 52^. Thus, the selection of the appropriate predator for clinical purposes would require larger and more in-depth studies on predation to overcome predation resistance in specific prey species and individual strains within the species.

## Materials and methods

### Strains and growth conditions

The *B. bacteriovorus* 109J wildtype strain was used as predator against 78 *P. aeruginosa* strains selected from previously well-characterized collections obtained from respiratory samples of CF patients (n = 30)^27^, and invasive BSI isolates (n = 48)^28^. All 78 *P. aeruginosa* isolates were collected and characterized in earlier studies, for which the appropriate ethical approval and consent were obtained^27, 28^. The selection of prey was based on representative sequence types (STs) determined by MLST, both frequent and rare STs, their antibiotic susceptibility phenotype, including multidrug-resistant isolates, as well as their morphology in CF isolates, differentiating mucoid (n = 7) and non-mucoid isolates (n = 23) (Tables S1 and S2). The methodologies used for the molecular characterization (ST, antibiotic resistance profile, morphotype) of *P. aeruginosa* collections were detailed in the previous publication^27, 28^.

The predation activity of the PHA-depolymerase mutant *B.* *bacteriovorus* bd2637^30^ was only tested against the 48 BSI isolates. Mutations were manually inspected with Artemis^53^ using the Genbank accession numbers: SRX10641169 and SRX10641170 for *B. bacteriovorus* 109J and the bd2637 mutant, respectively (Table S5).

Predators were routinely grown as previously described^54, 55^ by co-cultivation with *Pseudomonas putida* KT2440 instead of *P. aeruginosa* to prevent the bias that might result from possible adaptive influence on the growth of a particular ST type of *P. aeruginosa*. Diluted Nutrient Broth (DNB) (0.8 g/l nutrient broth at pH 7.4) was used to recover the predator cells from glycerol stocks and HEPES buffer (25 mM at pH 7.8) was used to perform the predation experiments. Both DNB and HEPES buffer were supplemented with 2 mM CaCl_2_·2H_2_O and 3 mM MgCl_2_·3H_2_O. To purify the predator, co-cultures were filtered twice through a 0.45-μm filter. Finally, *P. aeruginosa* strains were cultivated on Luria Broth (LB) for 16 h at 37 °C and were further diluted in HEPES buffer to an optical density at 600 nm of 1 (OD_600_ 1) for the subsequent predation experiments.

### Predation experiments

Prey (100 μl to final OD_600_ 0.3) and predator (100 μl) co-cultures were performed on 96-microwell plates for 24 h at 30 °C with orbital shaking in a Synergy HTX (BioTek) with cell density monitoring (OD_600_) each 15 min. Each prey was tested in at least 2–3 independent biological replicates, with the results expressed as mean values of these replicates for all experiments. Growth control wells without the predator were also included for each *P. aeruginosa* isolate.

To assure the homogeneity of the predator inoculum, each experiment had a parallel control co-culture of *B. bacteriovorus* (100 µl) and *P. putida* (100 µl of 10^9^ CFU/ml); and after incubation, prey survival was determined by seeding serial dilutions in agar plates, discarding those sets in which the viable prey decrease upon the control *P. putida* predation was different than 3 log10 and 4 log10 for wildtype and bd2637 predators, respectively, as previously described^30, 39^.

Thus, predation experiments included control at the beginning of the experiment of both predator and prey inoculum (viable counts corresponding to OD_600_ = 0.3). Viable cell count of the prey was performed at 0 h and 24 h of predation. 10 µl of the corresponding dilution of co-coculture (including the control without predator and the predation control with *P. putida*) was plated on LB solid medium and colony-forming units (CFU/ml) were counted after 24 h of incubation at 37 °C.

The predation activity of a predator upon a prey collection is defined by two parameters: (1) the intraspecies predation range (I-PR), which corresponds to the proportion of susceptible prey strains within a species collection, and (2) the predation efficiency (PE), specific for each prey/predator combination, which is determined as the ratio between viable prey cells after predator exposure compared to the initial inoculum. PE was expressed in log10 values. Positive predation was only considered when PE was > 0.5, corresponding with the predation of 50% of the population (*p* < 0.046, Table S1).

### Statistical analysis

The Mann–Whitney U test was used to compare PE values between CF and BSI collections, after assuming non-normality with the Shapiro–Wilk normality test. Additionally, associations between qualitative features of *Pseudomonas* collections and the quantitative variable I-PR were performed using simple linear model. The differences in I-PR between the CF and BSI collections were explored using the chi-squared test. All data sets were analyzed using R version 3.5.0, and plotting was performed using ggplot version 2.2.1. and RStudio software v.1.2.5001 (R Core Team (2019). R: A language and environment for statistical computing. R Foundation for Statistical Computing, Vienna, Austria. URL https://www.R-project.org/).

## Supplementary Information

Supplementary Information.
