# Supplementary material for: Strain-specific predation of Bdellovibrio bacteriovorus on Pseudomonas aeruginosa with a higher range for cystic fibrosis than for bacteremia isolates
Source: Sci Rep. 2022 Jun 22;12:10523. doi: 10.1038/s41598-022-14378-5 (PMC9217795; doi:10.1038/s41598-022-14378-5)
Supplement: Supplementary file 1 — Supplementary Information. [file 41598_2022_14378_MOESM1_ESM.pptx]

## Slide 1
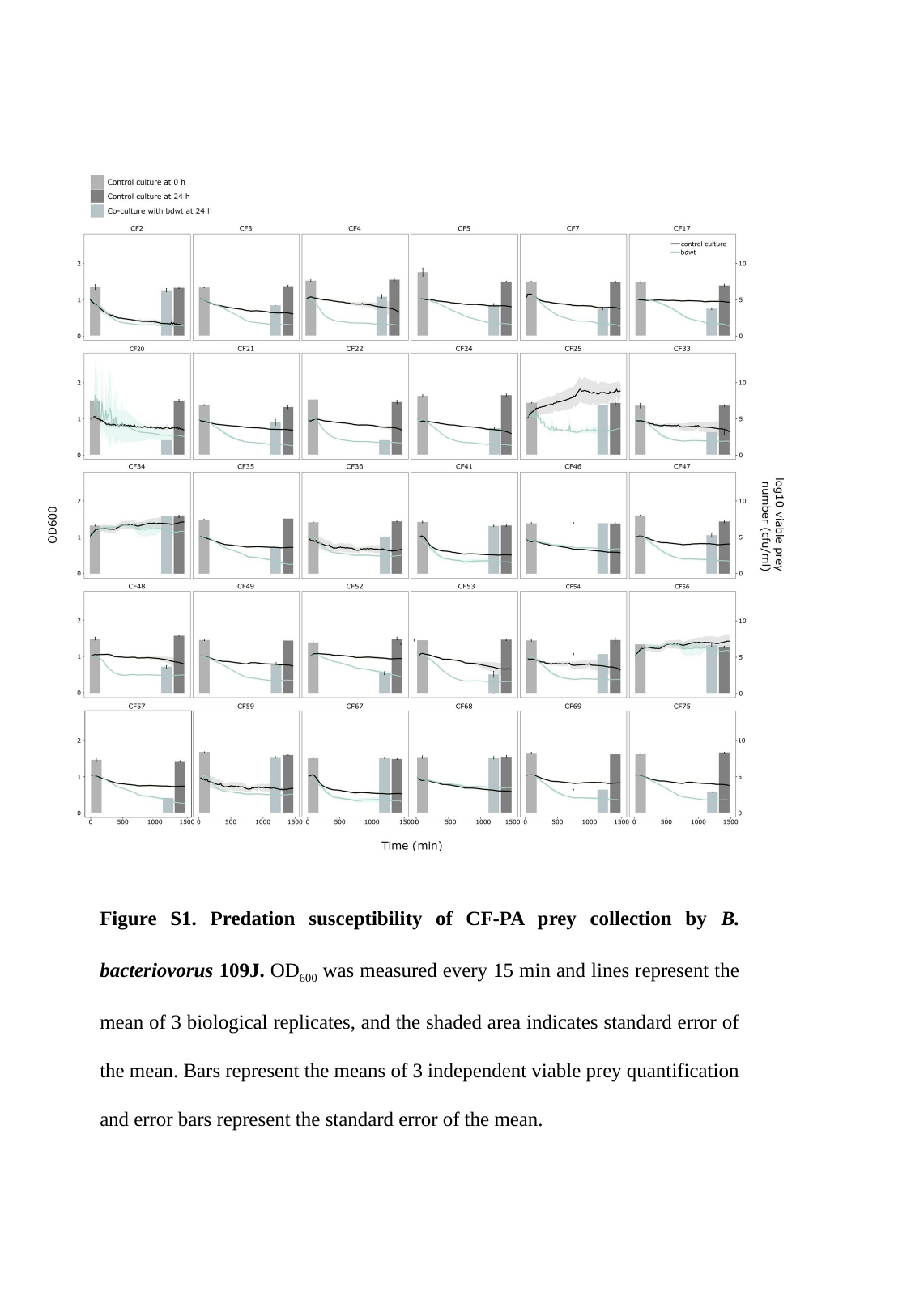

Figure S1. Predation susceptibility of CF-PA prey collection by B. bacteriovorus 109J. OD600 was measured every 15 min and lines represent the mean of 3 biological replicates, and the shaded area indicates standard error of the mean. Bars represent the means of 3 independent viable prey quantification and error bars represent the standard error of the mean.

## Slide 2
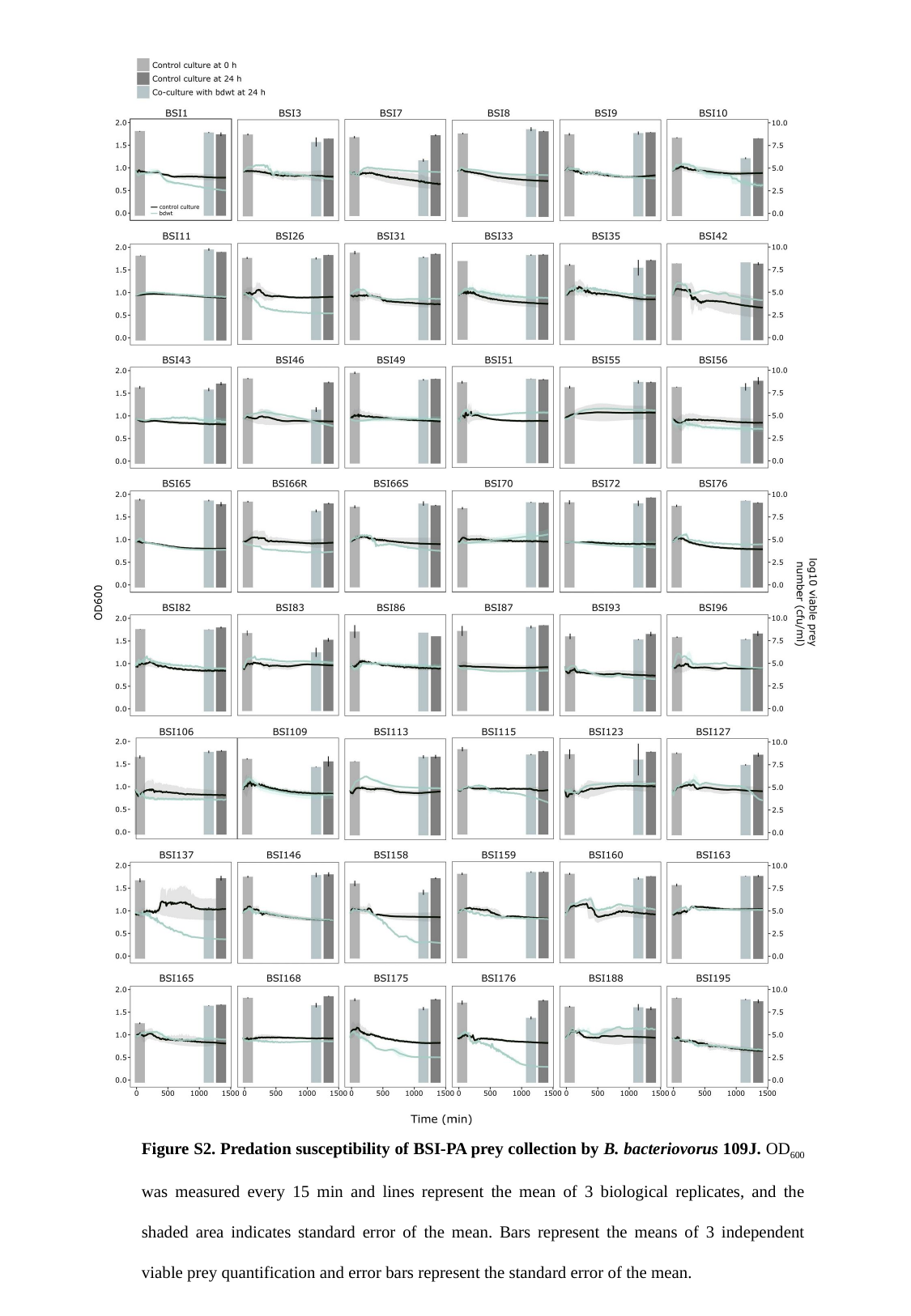

Figure S2. Predation susceptibility of BSI-PA prey collection by B. bacteriovorus 109J. OD600 was measured every 15 min and lines represent the mean of 3 biological replicates, and the shaded area indicates standard error of the mean. Bars represent the means of 3 independent viable prey quantification and error bars represent the standard error of the mean.

## Slide 3
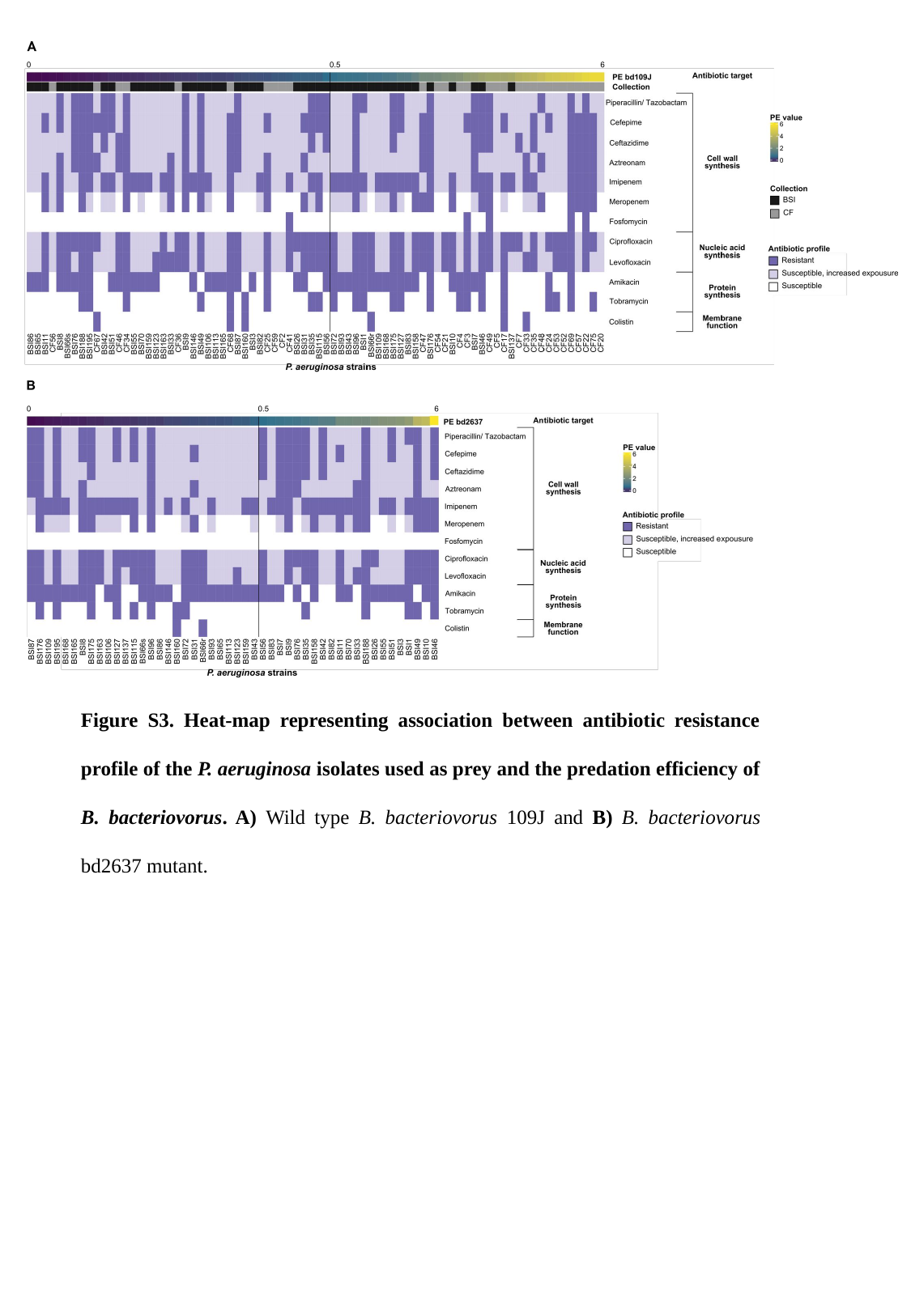

Figure S3. Heat-map representing association between antibiotic resistance profile of the P. aeruginosa isolates used as prey and the predation efficiency of B. bacteriovorus. A) Wild type B. bacteriovorus 109J and B) B. bacteriovorus bd2637 mutant.

## Slide 4
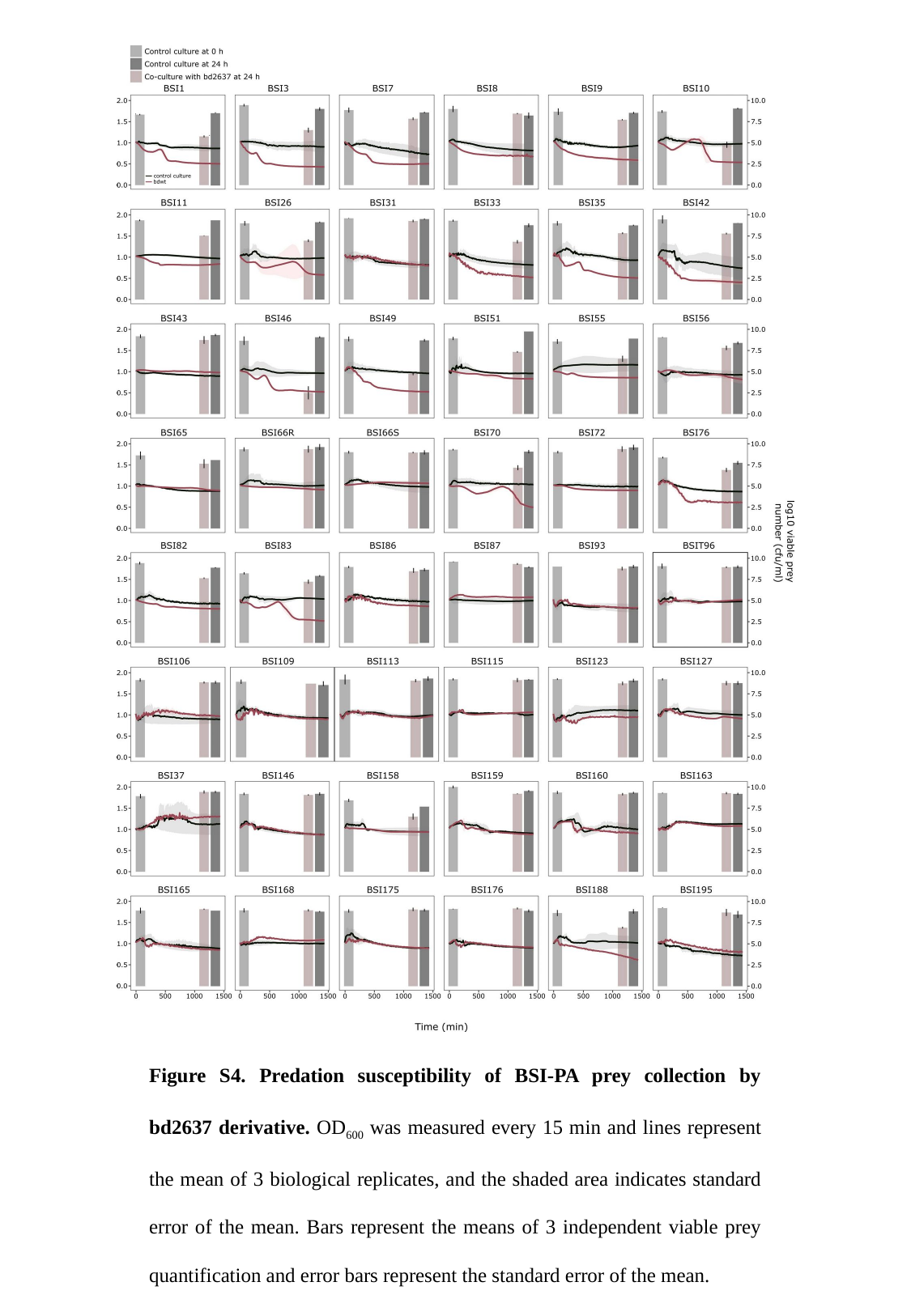

Figure S4. Predation susceptibility of BSI-PA prey collection by bd2637 derivative. OD600 was measured every 15 min and lines represent the mean of 3 biological replicates, and the shaded area indicates standard error of the mean. Bars represent the means of 3 independent viable prey quantification and error bars represent the standard error of the mean.

## Slide 5
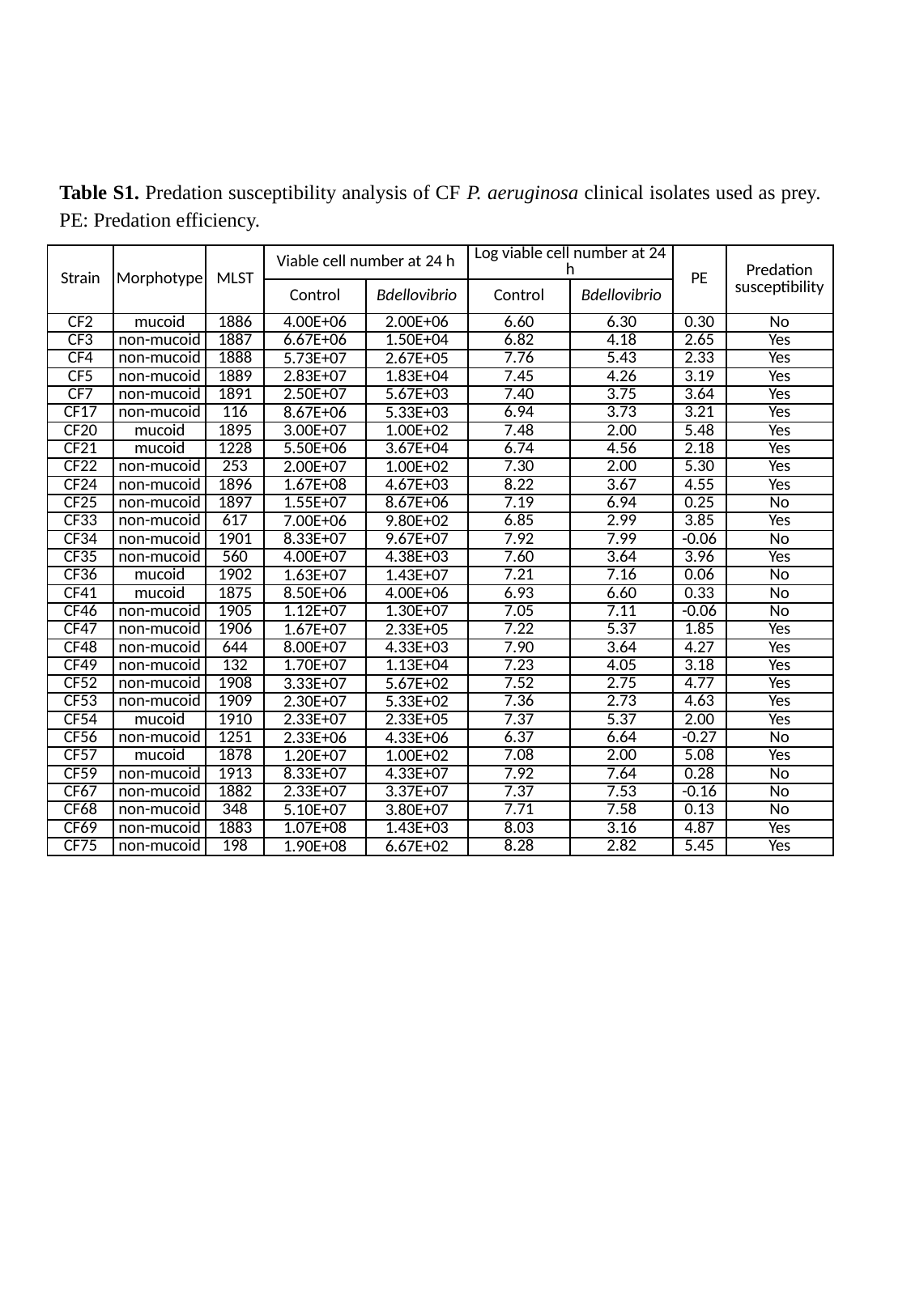

Table S1. Predation susceptibility analysis of CF P. aeruginosa clinical isolates used as prey. PE: Predation efficiency.
| Strain | Morphotype | MLST | Viable cell number at 24 h | | Log viable cell number at 24 h | | PE | Predation susceptibility |
| --- | --- | --- | --- | --- | --- | --- | --- | --- |
| | | | Control | Bdellovibrio | Control | Bdellovibrio | | |
| CF2 | mucoid | 1886 | 4.00E+06 | 2.00E+06 | 6.60 | 6.30 | 0.30 | No |
| CF3 | non-mucoid | 1887 | 6.67E+06 | 1.50E+04 | 6.82 | 4.18 | 2.65 | Yes |
| CF4 | non-mucoid | 1888 | 5.73E+07 | 2.67E+05 | 7.76 | 5.43 | 2.33 | Yes |
| CF5 | non-mucoid | 1889 | 2.83E+07 | 1.83E+04 | 7.45 | 4.26 | 3.19 | Yes |
| CF7 | non-mucoid | 1891 | 2.50E+07 | 5.67E+03 | 7.40 | 3.75 | 3.64 | Yes |
| CF17 | non-mucoid | 116 | 8.67E+06 | 5.33E+03 | 6.94 | 3.73 | 3.21 | Yes |
| CF20 | mucoid | 1895 | 3.00E+07 | 1.00E+02 | 7.48 | 2.00 | 5.48 | Yes |
| CF21 | mucoid | 1228 | 5.50E+06 | 3.67E+04 | 6.74 | 4.56 | 2.18 | Yes |
| CF22 | non-mucoid | 253 | 2.00E+07 | 1.00E+02 | 7.30 | 2.00 | 5.30 | Yes |
| CF24 | non-mucoid | 1896 | 1.67E+08 | 4.67E+03 | 8.22 | 3.67 | 4.55 | Yes |
| CF25 | non-mucoid | 1897 | 1.55E+07 | 8.67E+06 | 7.19 | 6.94 | 0.25 | No |
| CF33 | non-mucoid | 617 | 7.00E+06 | 9.80E+02 | 6.85 | 2.99 | 3.85 | Yes |
| CF34 | non-mucoid | 1901 | 8.33E+07 | 9.67E+07 | 7.92 | 7.99 | -0.06 | No |
| CF35 | non-mucoid | 560 | 4.00E+07 | 4.38E+03 | 7.60 | 3.64 | 3.96 | Yes |
| CF36 | mucoid | 1902 | 1.63E+07 | 1.43E+07 | 7.21 | 7.16 | 0.06 | No |
| CF41 | mucoid | 1875 | 8.50E+06 | 4.00E+06 | 6.93 | 6.60 | 0.33 | No |
| CF46 | non-mucoid | 1905 | 1.12E+07 | 1.30E+07 | 7.05 | 7.11 | -0.06 | No |
| CF47 | non-mucoid | 1906 | 1.67E+07 | 2.33E+05 | 7.22 | 5.37 | 1.85 | Yes |
| CF48 | non-mucoid | 644 | 8.00E+07 | 4.33E+03 | 7.90 | 3.64 | 4.27 | Yes |
| CF49 | non-mucoid | 132 | 1.70E+07 | 1.13E+04 | 7.23 | 4.05 | 3.18 | Yes |
| CF52 | non-mucoid | 1908 | 3.33E+07 | 5.67E+02 | 7.52 | 2.75 | 4.77 | Yes |
| CF53 | non-mucoid | 1909 | 2.30E+07 | 5.33E+02 | 7.36 | 2.73 | 4.63 | Yes |
| CF54 | mucoid | 1910 | 2.33E+07 | 2.33E+05 | 7.37 | 5.37 | 2.00 | Yes |
| CF56 | non-mucoid | 1251 | 2.33E+06 | 4.33E+06 | 6.37 | 6.64 | -0.27 | No |
| CF57 | mucoid | 1878 | 1.20E+07 | 1.00E+02 | 7.08 | 2.00 | 5.08 | Yes |
| CF59 | non-mucoid | 1913 | 8.33E+07 | 4.33E+07 | 7.92 | 7.64 | 0.28 | No |
| CF67 | non-mucoid | 1882 | 2.33E+07 | 3.37E+07 | 7.37 | 7.53 | -0.16 | No |
| CF68 | non-mucoid | 348 | 5.10E+07 | 3.80E+07 | 7.71 | 7.58 | 0.13 | No |
| CF69 | non-mucoid | 1883 | 1.07E+08 | 1.43E+03 | 8.03 | 3.16 | 4.87 | Yes |
| CF75 | non-mucoid | 198 | 1.90E+08 | 6.67E+02 | 8.28 | 2.82 | 5.45 | Yes |

## Slide 6
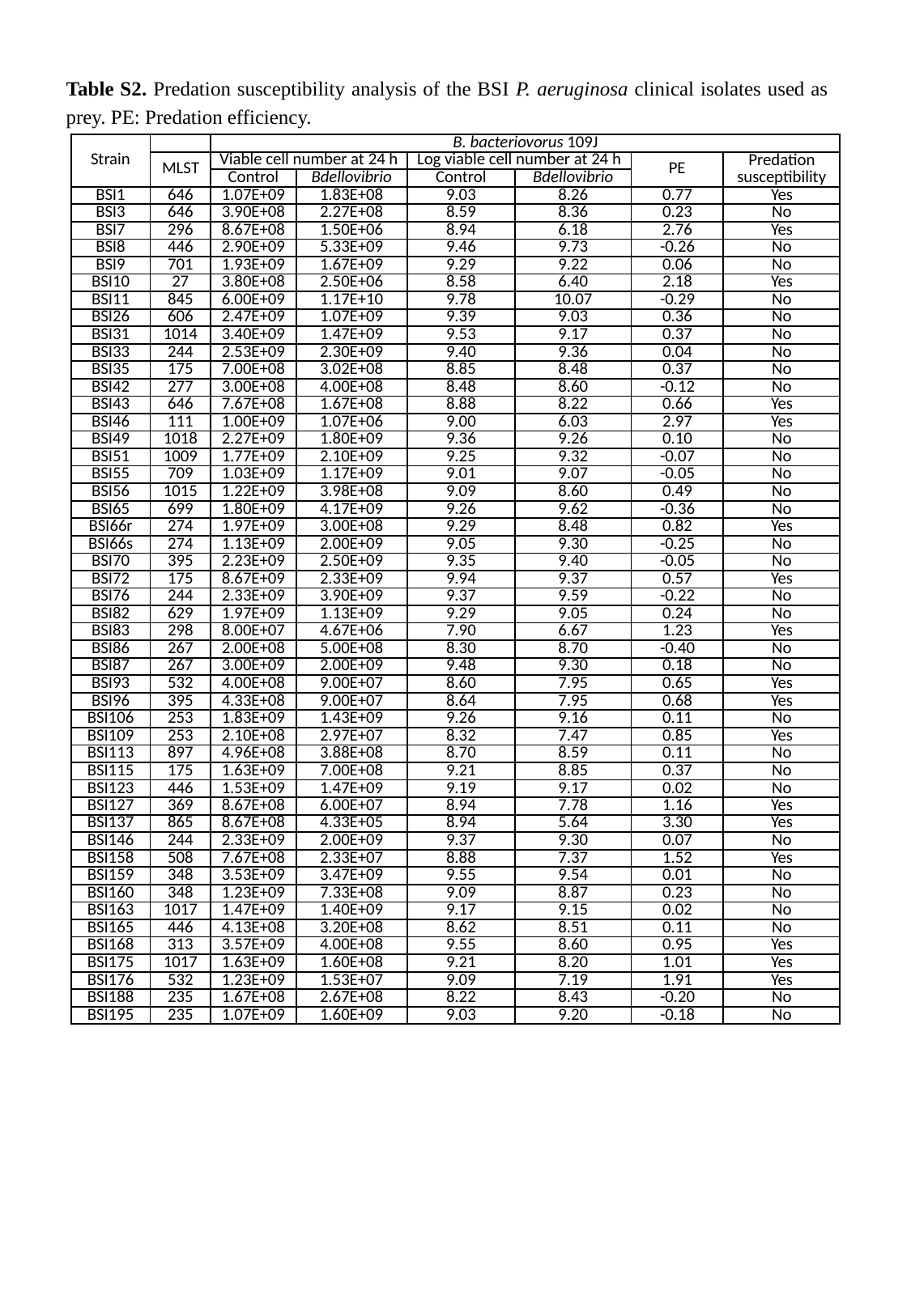

Table S2. Predation susceptibility analysis of the BSI P. aeruginosa clinical isolates used as prey. PE: Predation efficiency.
| Strain | | B. bacteriovorus 109J | | | | | |
| --- | --- | --- | --- | --- | --- | --- | --- |
| | MLST | Viable cell number at 24 h | | Log viable cell number at 24 h | | PE | Predation susceptibility |
| | | Control | Bdellovibrio | Control | Bdellovibrio | | |
| BSI1 | 646 | 1.07E+09 | 1.83E+08 | 9.03 | 8.26 | 0.77 | Yes |
| BSI3 | 646 | 3.90E+08 | 2.27E+08 | 8.59 | 8.36 | 0.23 | No |
| BSI7 | 296 | 8.67E+08 | 1.50E+06 | 8.94 | 6.18 | 2.76 | Yes |
| BSI8 | 446 | 2.90E+09 | 5.33E+09 | 9.46 | 9.73 | -0.26 | No |
| BSI9 | 701 | 1.93E+09 | 1.67E+09 | 9.29 | 9.22 | 0.06 | No |
| BSI10 | 27 | 3.80E+08 | 2.50E+06 | 8.58 | 6.40 | 2.18 | Yes |
| BSI11 | 845 | 6.00E+09 | 1.17E+10 | 9.78 | 10.07 | -0.29 | No |
| BSI26 | 606 | 2.47E+09 | 1.07E+09 | 9.39 | 9.03 | 0.36 | No |
| BSI31 | 1014 | 3.40E+09 | 1.47E+09 | 9.53 | 9.17 | 0.37 | No |
| BSI33 | 244 | 2.53E+09 | 2.30E+09 | 9.40 | 9.36 | 0.04 | No |
| BSI35 | 175 | 7.00E+08 | 3.02E+08 | 8.85 | 8.48 | 0.37 | No |
| BSI42 | 277 | 3.00E+08 | 4.00E+08 | 8.48 | 8.60 | -0.12 | No |
| BSI43 | 646 | 7.67E+08 | 1.67E+08 | 8.88 | 8.22 | 0.66 | Yes |
| BSI46 | 111 | 1.00E+09 | 1.07E+06 | 9.00 | 6.03 | 2.97 | Yes |
| BSI49 | 1018 | 2.27E+09 | 1.80E+09 | 9.36 | 9.26 | 0.10 | No |
| BSI51 | 1009 | 1.77E+09 | 2.10E+09 | 9.25 | 9.32 | -0.07 | No |
| BSI55 | 709 | 1.03E+09 | 1.17E+09 | 9.01 | 9.07 | -0.05 | No |
| BSI56 | 1015 | 1.22E+09 | 3.98E+08 | 9.09 | 8.60 | 0.49 | No |
| BSI65 | 699 | 1.80E+09 | 4.17E+09 | 9.26 | 9.62 | -0.36 | No |
| BSI66r | 274 | 1.97E+09 | 3.00E+08 | 9.29 | 8.48 | 0.82 | Yes |
| BSI66s | 274 | 1.13E+09 | 2.00E+09 | 9.05 | 9.30 | -0.25 | No |
| BSI70 | 395 | 2.23E+09 | 2.50E+09 | 9.35 | 9.40 | -0.05 | No |
| BSI72 | 175 | 8.67E+09 | 2.33E+09 | 9.94 | 9.37 | 0.57 | Yes |
| BSI76 | 244 | 2.33E+09 | 3.90E+09 | 9.37 | 9.59 | -0.22 | No |
| BSI82 | 629 | 1.97E+09 | 1.13E+09 | 9.29 | 9.05 | 0.24 | No |
| BSI83 | 298 | 8.00E+07 | 4.67E+06 | 7.90 | 6.67 | 1.23 | Yes |
| BSI86 | 267 | 2.00E+08 | 5.00E+08 | 8.30 | 8.70 | -0.40 | No |
| BSI87 | 267 | 3.00E+09 | 2.00E+09 | 9.48 | 9.30 | 0.18 | No |
| BSI93 | 532 | 4.00E+08 | 9.00E+07 | 8.60 | 7.95 | 0.65 | Yes |
| BSI96 | 395 | 4.33E+08 | 9.00E+07 | 8.64 | 7.95 | 0.68 | Yes |
| BSI106 | 253 | 1.83E+09 | 1.43E+09 | 9.26 | 9.16 | 0.11 | No |
| BSI109 | 253 | 2.10E+08 | 2.97E+07 | 8.32 | 7.47 | 0.85 | Yes |
| BSI113 | 897 | 4.96E+08 | 3.88E+08 | 8.70 | 8.59 | 0.11 | No |
| BSI115 | 175 | 1.63E+09 | 7.00E+08 | 9.21 | 8.85 | 0.37 | No |
| BSI123 | 446 | 1.53E+09 | 1.47E+09 | 9.19 | 9.17 | 0.02 | No |
| BSI127 | 369 | 8.67E+08 | 6.00E+07 | 8.94 | 7.78 | 1.16 | Yes |
| BSI137 | 865 | 8.67E+08 | 4.33E+05 | 8.94 | 5.64 | 3.30 | Yes |
| BSI146 | 244 | 2.33E+09 | 2.00E+09 | 9.37 | 9.30 | 0.07 | No |
| BSI158 | 508 | 7.67E+08 | 2.33E+07 | 8.88 | 7.37 | 1.52 | Yes |
| BSI159 | 348 | 3.53E+09 | 3.47E+09 | 9.55 | 9.54 | 0.01 | No |
| BSI160 | 348 | 1.23E+09 | 7.33E+08 | 9.09 | 8.87 | 0.23 | No |
| BSI163 | 1017 | 1.47E+09 | 1.40E+09 | 9.17 | 9.15 | 0.02 | No |
| BSI165 | 446 | 4.13E+08 | 3.20E+08 | 8.62 | 8.51 | 0.11 | No |
| BSI168 | 313 | 3.57E+09 | 4.00E+08 | 9.55 | 8.60 | 0.95 | Yes |
| BSI175 | 1017 | 1.63E+09 | 1.60E+08 | 9.21 | 8.20 | 1.01 | Yes |
| BSI176 | 532 | 1.23E+09 | 1.53E+07 | 9.09 | 7.19 | 1.91 | Yes |
| BSI188 | 235 | 1.67E+08 | 2.67E+08 | 8.22 | 8.43 | -0.20 | No |
| BSI195 | 235 | 1.07E+09 | 1.60E+09 | 9.03 | 9.20 | -0.18 | No |

## Slide 7
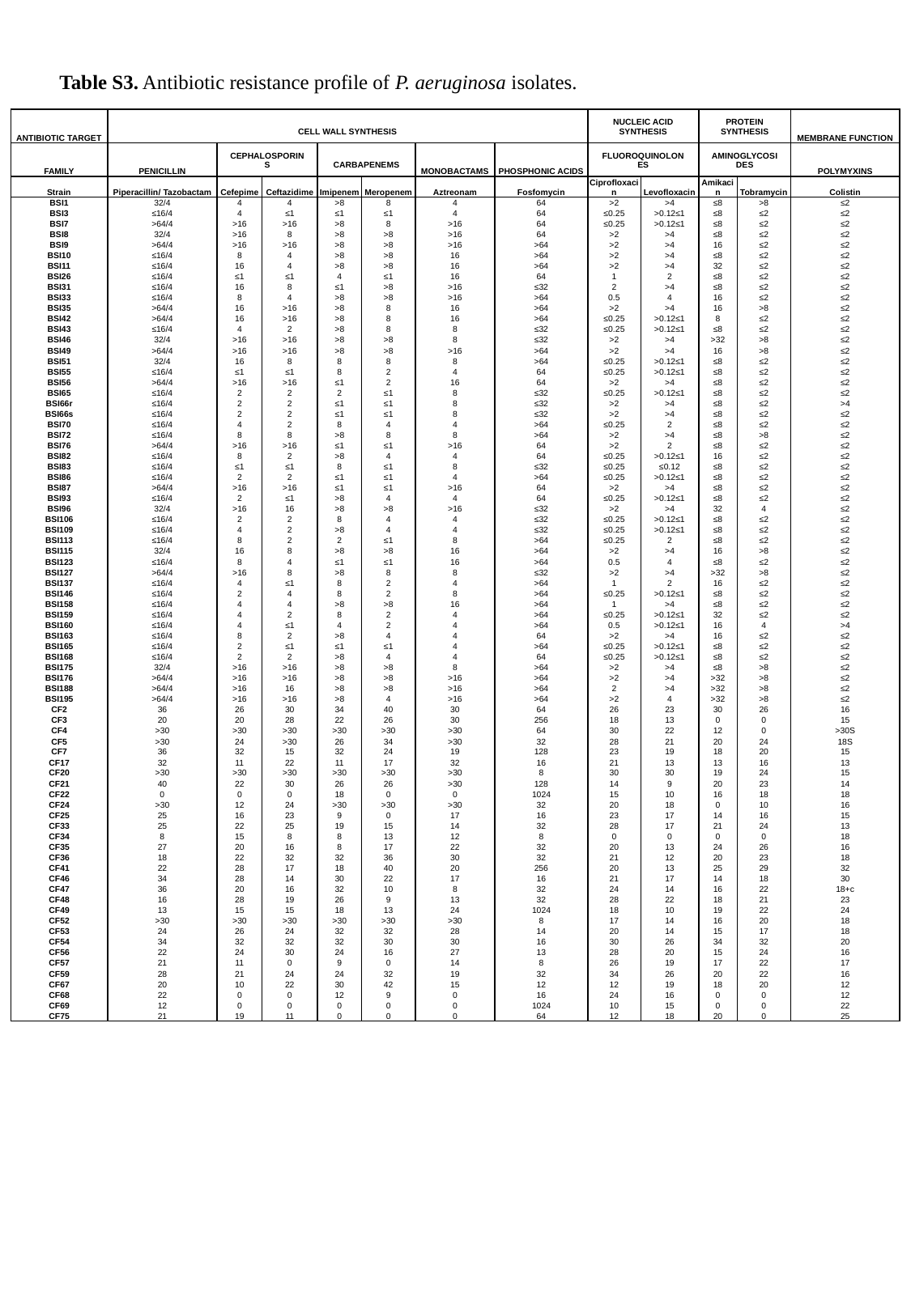

Table S3. Antibiotic resistance profile of P. aeruginosa isolates.
| ANTIBIOTIC TARGET | CELL WALL SYNTHESIS | | | | | | | NUCLEIC ACID SYNTHESIS | | PROTEIN SYNTHESIS | | MEMBRANE FUNCTION |
| --- | --- | --- | --- | --- | --- | --- | --- | --- | --- | --- | --- | --- |
| FAMILY | PENICILLIN | CEPHALOSPORINS | | CARBAPENEMS | | MONOBACTAMS | PHOSPHONIC ACIDS | FLUOROQUINOLONES | | AMINOGLYCOSIDES | | POLYMYXINS |
| Strain | Piperacillin/ Tazobactam | Cefepime | Ceftazidime | Imipenem | Meropenem | Aztreonam | Fosfomycin | Ciprofloxacin | Levofloxacin | Amikacin | Tobramycin | Colistin |
| BSI1 | 32/4 | 4 | 4 | >8 | 8 | 4 | 64 | >2 | >4 | ≤8 | >8 | ≤2 |
| BSI3 | ≤16/4 | 4 | ≤1 | ≤1 | ≤1 | 4 | 64 | ≤0.25 | >0.12≤1 | ≤8 | ≤2 | ≤2 |
| BSI7 | >64/4 | >16 | >16 | >8 | 8 | >16 | 64 | ≤0.25 | >0.12≤1 | ≤8 | ≤2 | ≤2 |
| BSI8 | 32/4 | >16 | 8 | >8 | >8 | >16 | 64 | >2 | >4 | ≤8 | ≤2 | ≤2 |
| BSI9 | >64/4 | >16 | >16 | >8 | >8 | >16 | >64 | >2 | >4 | 16 | ≤2 | ≤2 |
| BSI10 | ≤16/4 | 8 | 4 | >8 | >8 | 16 | >64 | >2 | >4 | ≤8 | ≤2 | ≤2 |
| BSI11 | ≤16/4 | 16 | 4 | >8 | >8 | 16 | >64 | >2 | >4 | 32 | ≤2 | ≤2 |
| BSI26 | ≤16/4 | ≤1 | ≤1 | 4 | ≤1 | 16 | 64 | 1 | 2 | ≤8 | ≤2 | ≤2 |
| BSI31 | ≤16/4 | 16 | 8 | ≤1 | >8 | >16 | ≤32 | 2 | >4 | ≤8 | ≤2 | ≤2 |
| BSI33 | ≤16/4 | 8 | 4 | >8 | >8 | >16 | >64 | 0.5 | 4 | 16 | ≤2 | ≤2 |
| BSI35 | >64/4 | 16 | >16 | >8 | 8 | 16 | >64 | >2 | >4 | 16 | >8 | ≤2 |
| BSI42 | >64/4 | 16 | >16 | >8 | 8 | 16 | >64 | ≤0.25 | >0.12≤1 | 8 | ≤2 | ≤2 |
| BSI43 | ≤16/4 | 4 | 2 | >8 | 8 | 8 | ≤32 | ≤0.25 | >0.12≤1 | ≤8 | ≤2 | ≤2 |
| BSI46 | 32/4 | >16 | >16 | >8 | >8 | 8 | ≤32 | >2 | >4 | >32 | >8 | ≤2 |
| BSI49 | >64/4 | >16 | >16 | >8 | >8 | >16 | >64 | >2 | >4 | 16 | >8 | ≤2 |
| BSI51 | 32/4 | 16 | 8 | 8 | 8 | 8 | >64 | ≤0.25 | >0.12≤1 | ≤8 | ≤2 | ≤2 |
| BSI55 | ≤16/4 | ≤1 | ≤1 | 8 | 2 | 4 | 64 | ≤0.25 | >0.12≤1 | ≤8 | ≤2 | ≤2 |
| BSI56 | >64/4 | >16 | >16 | ≤1 | 2 | 16 | 64 | >2 | >4 | ≤8 | ≤2 | ≤2 |
| BSI65 | ≤16/4 | 2 | 2 | 2 | ≤1 | 8 | ≤32 | ≤0.25 | >0.12≤1 | ≤8 | ≤2 | ≤2 |
| BSI66r | ≤16/4 | 2 | 2 | ≤1 | ≤1 | 8 | ≤32 | >2 | >4 | ≤8 | ≤2 | >4 |
| BSI66s | ≤16/4 | 2 | 2 | ≤1 | ≤1 | 8 | ≤32 | >2 | >4 | ≤8 | ≤2 | ≤2 |
| BSI70 | ≤16/4 | 4 | 2 | 8 | 4 | 4 | >64 | ≤0.25 | 2 | ≤8 | ≤2 | ≤2 |
| BSI72 | ≤16/4 | 8 | 8 | >8 | 8 | 8 | >64 | >2 | >4 | ≤8 | >8 | ≤2 |
| BSI76 | >64/4 | >16 | >16 | ≤1 | ≤1 | >16 | 64 | >2 | 2 | ≤8 | ≤2 | ≤2 |
| BSI82 | ≤16/4 | 8 | 2 | >8 | 4 | 4 | 64 | ≤0.25 | >0.12≤1 | 16 | ≤2 | ≤2 |
| BSI83 | ≤16/4 | ≤1 | ≤1 | 8 | ≤1 | 8 | ≤32 | ≤0.25 | ≤0.12 | ≤8 | ≤2 | ≤2 |
| BSI86 | ≤16/4 | 2 | 2 | ≤1 | ≤1 | 4 | >64 | ≤0.25 | >0.12≤1 | ≤8 | ≤2 | ≤2 |
| BSI87 | >64/4 | >16 | >16 | ≤1 | ≤1 | >16 | 64 | >2 | >4 | ≤8 | ≤2 | ≤2 |
| BSI93 | ≤16/4 | 2 | ≤1 | >8 | 4 | 4 | 64 | ≤0.25 | >0.12≤1 | ≤8 | ≤2 | ≤2 |
| BSI96 | 32/4 | >16 | 16 | >8 | >8 | >16 | ≤32 | >2 | >4 | 32 | 4 | ≤2 |
| BSI106 | ≤16/4 | 2 | 2 | 8 | 4 | 4 | ≤32 | ≤0.25 | >0.12≤1 | ≤8 | ≤2 | ≤2 |
| BSI109 | ≤16/4 | 4 | 2 | >8 | 4 | 4 | ≤32 | ≤0.25 | >0.12≤1 | ≤8 | ≤2 | ≤2 |
| BSI113 | ≤16/4 | 8 | 2 | 2 | ≤1 | 8 | >64 | ≤0.25 | 2 | ≤8 | ≤2 | ≤2 |
| BSI115 | 32/4 | 16 | 8 | >8 | >8 | 16 | >64 | >2 | >4 | 16 | >8 | ≤2 |
| BSI123 | ≤16/4 | 8 | 4 | ≤1 | ≤1 | 16 | >64 | 0.5 | 4 | ≤8 | ≤2 | ≤2 |
| BSI127 | >64/4 | >16 | 8 | >8 | 8 | 8 | ≤32 | >2 | >4 | >32 | >8 | ≤2 |
| BSI137 | ≤16/4 | 4 | ≤1 | 8 | 2 | 4 | >64 | 1 | 2 | 16 | ≤2 | ≤2 |
| BSI146 | ≤16/4 | 2 | 4 | 8 | 2 | 8 | >64 | ≤0.25 | >0.12≤1 | ≤8 | ≤2 | ≤2 |
| BSI158 | ≤16/4 | 4 | 4 | >8 | >8 | 16 | >64 | 1 | >4 | ≤8 | ≤2 | ≤2 |
| BSI159 | ≤16/4 | 4 | 2 | 8 | 2 | 4 | >64 | ≤0.25 | >0.12≤1 | 32 | ≤2 | ≤2 |
| BSI160 | ≤16/4 | 4 | ≤1 | 4 | 2 | 4 | >64 | 0.5 | >0.12≤1 | 16 | 4 | >4 |
| BSI163 | ≤16/4 | 8 | 2 | >8 | 4 | 4 | 64 | >2 | >4 | 16 | ≤2 | ≤2 |
| BSI165 | ≤16/4 | 2 | ≤1 | ≤1 | ≤1 | 4 | >64 | ≤0.25 | >0.12≤1 | ≤8 | ≤2 | ≤2 |
| BSI168 | ≤16/4 | 2 | 2 | >8 | 4 | 4 | 64 | ≤0.25 | >0.12≤1 | ≤8 | ≤2 | ≤2 |
| BSI175 | 32/4 | >16 | >16 | >8 | >8 | 8 | >64 | >2 | >4 | ≤8 | >8 | ≤2 |
| BSI176 | >64/4 | >16 | >16 | >8 | >8 | >16 | >64 | >2 | >4 | >32 | >8 | ≤2 |
| BSI188 | >64/4 | >16 | 16 | >8 | >8 | >16 | >64 | 2 | >4 | >32 | >8 | ≤2 |
| BSI195 | >64/4 | >16 | >16 | >8 | 4 | >16 | >64 | >2 | 4 | >32 | >8 | ≤2 |
| CF2 | 36 | 26 | 30 | 34 | 40 | 30 | 64 | 26 | 23 | 30 | 26 | 16 |
| CF3 | 20 | 20 | 28 | 22 | 26 | 30 | 256 | 18 | 13 | 0 | 0 | 15 |
| CF4 | >30 | >30 | >30 | >30 | >30 | >30 | 64 | 30 | 22 | 12 | 0 | >30S |
| CF5 | >30 | 24 | >30 | 26 | 34 | >30 | 32 | 28 | 21 | 20 | 24 | 18S |
| CF7 | 36 | 32 | 15 | 32 | 24 | 19 | 128 | 23 | 19 | 18 | 20 | 15 |
| CF17 | 32 | 11 | 22 | 11 | 17 | 32 | 16 | 21 | 13 | 13 | 16 | 13 |
| CF20 | >30 | >30 | >30 | >30 | >30 | >30 | 8 | 30 | 30 | 19 | 24 | 15 |
| CF21 | 40 | 22 | 30 | 26 | 26 | >30 | 128 | 14 | 9 | 20 | 23 | 14 |
| CF22 | 0 | 0 | 0 | 18 | 0 | 0 | 1024 | 15 | 10 | 16 | 18 | 18 |
| CF24 | >30 | 12 | 24 | >30 | >30 | >30 | 32 | 20 | 18 | 0 | 10 | 16 |
| CF25 | 25 | 16 | 23 | 9 | 0 | 17 | 16 | 23 | 17 | 14 | 16 | 15 |
| CF33 | 25 | 22 | 25 | 19 | 15 | 14 | 32 | 28 | 17 | 21 | 24 | 13 |
| CF34 | 8 | 15 | 8 | 8 | 13 | 12 | 8 | 0 | 0 | 0 | 0 | 18 |
| CF35 | 27 | 20 | 16 | 8 | 17 | 22 | 32 | 20 | 13 | 24 | 26 | 16 |
| CF36 | 18 | 22 | 32 | 32 | 36 | 30 | 32 | 21 | 12 | 20 | 23 | 18 |
| CF41 | 22 | 28 | 17 | 18 | 40 | 20 | 256 | 20 | 13 | 25 | 29 | 32 |
| CF46 | 34 | 28 | 14 | 30 | 22 | 17 | 16 | 21 | 17 | 14 | 18 | 30 |
| CF47 | 36 | 20 | 16 | 32 | 10 | 8 | 32 | 24 | 14 | 16 | 22 | 18+c |
| CF48 | 16 | 28 | 19 | 26 | 9 | 13 | 32 | 28 | 22 | 18 | 21 | 23 |
| CF49 | 13 | 15 | 15 | 18 | 13 | 24 | 1024 | 18 | 10 | 19 | 22 | 24 |
| CF52 | >30 | >30 | >30 | >30 | >30 | >30 | 8 | 17 | 14 | 16 | 20 | 18 |
| CF53 | 24 | 26 | 24 | 32 | 32 | 28 | 14 | 20 | 14 | 15 | 17 | 18 |
| CF54 | 34 | 32 | 32 | 32 | 30 | 30 | 16 | 30 | 26 | 34 | 32 | 20 |
| CF56 | 22 | 24 | 30 | 24 | 16 | 27 | 13 | 28 | 20 | 15 | 24 | 16 |
| CF57 | 21 | 11 | 0 | 9 | 0 | 14 | 8 | 26 | 19 | 17 | 22 | 17 |
| CF59 | 28 | 21 | 24 | 24 | 32 | 19 | 32 | 34 | 26 | 20 | 22 | 16 |
| CF67 | 20 | 10 | 22 | 30 | 42 | 15 | 12 | 12 | 19 | 18 | 20 | 12 |
| CF68 | 22 | 0 | 0 | 12 | 9 | 0 | 16 | 24 | 16 | 0 | 0 | 12 |
| CF69 | 12 | 0 | 0 | 0 | 0 | 0 | 1024 | 10 | 15 | 0 | 0 | 22 |
| CF75 | 21 | 19 | 11 | 0 | 0 | 0 | 64 | 12 | 18 | 20 | 0 | 25 |

## Slide 8
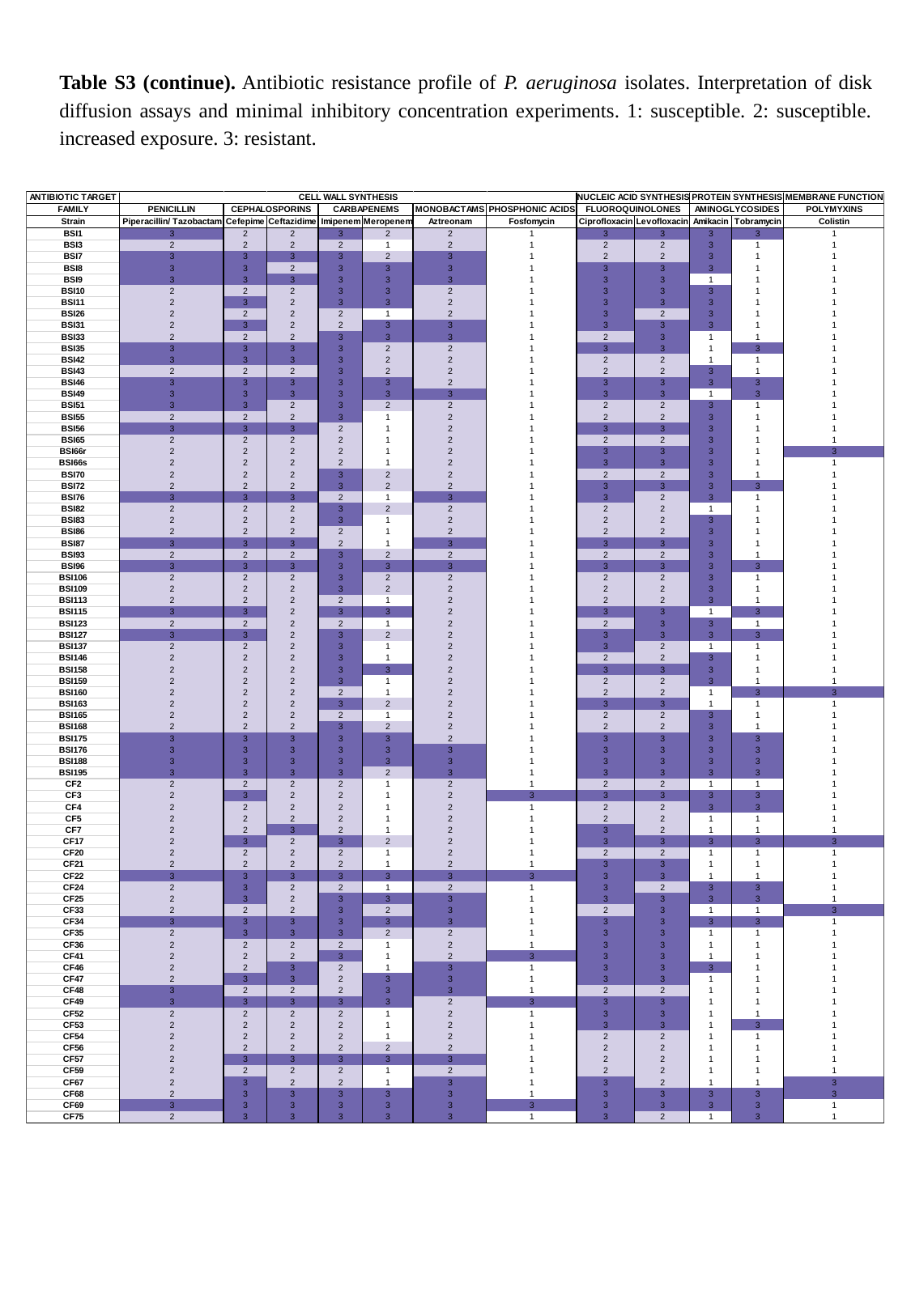

Table S3 (continue). Antibiotic resistance profile of P. aeruginosa isolates. Interpretation of disk diffusion assays and minimal inhibitory concentration experiments. 1: susceptible. 2: susceptible. increased exposure. 3: resistant.

## Slide 9
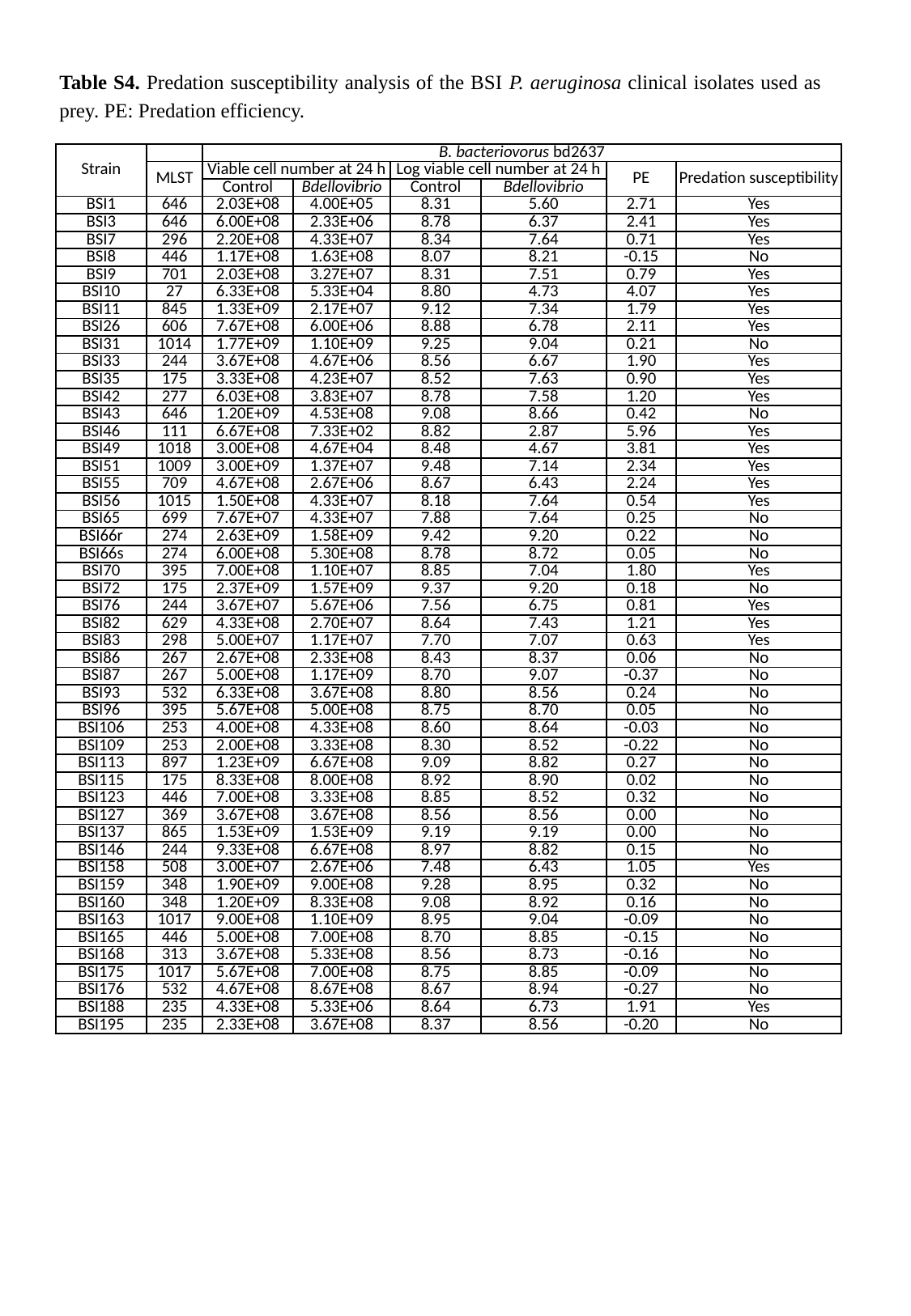

Table S4. Predation susceptibility analysis of the BSI P. aeruginosa clinical isolates used as prey. PE: Predation efficiency.
| Strain | | B. bacteriovorus bd2637 | | | | | |
| --- | --- | --- | --- | --- | --- | --- | --- |
| | MLST | Viable cell number at 24 h | | Log viable cell number at 24 h | | PE | Predation susceptibility |
| | | Control | Bdellovibrio | Control | Bdellovibrio | | |
| BSI1 | 646 | 2.03E+08 | 4.00E+05 | 8.31 | 5.60 | 2.71 | Yes |
| BSI3 | 646 | 6.00E+08 | 2.33E+06 | 8.78 | 6.37 | 2.41 | Yes |
| BSI7 | 296 | 2.20E+08 | 4.33E+07 | 8.34 | 7.64 | 0.71 | Yes |
| BSI8 | 446 | 1.17E+08 | 1.63E+08 | 8.07 | 8.21 | -0.15 | No |
| BSI9 | 701 | 2.03E+08 | 3.27E+07 | 8.31 | 7.51 | 0.79 | Yes |
| BSI10 | 27 | 6.33E+08 | 5.33E+04 | 8.80 | 4.73 | 4.07 | Yes |
| BSI11 | 845 | 1.33E+09 | 2.17E+07 | 9.12 | 7.34 | 1.79 | Yes |
| BSI26 | 606 | 7.67E+08 | 6.00E+06 | 8.88 | 6.78 | 2.11 | Yes |
| BSI31 | 1014 | 1.77E+09 | 1.10E+09 | 9.25 | 9.04 | 0.21 | No |
| BSI33 | 244 | 3.67E+08 | 4.67E+06 | 8.56 | 6.67 | 1.90 | Yes |
| BSI35 | 175 | 3.33E+08 | 4.23E+07 | 8.52 | 7.63 | 0.90 | Yes |
| BSI42 | 277 | 6.03E+08 | 3.83E+07 | 8.78 | 7.58 | 1.20 | Yes |
| BSI43 | 646 | 1.20E+09 | 4.53E+08 | 9.08 | 8.66 | 0.42 | No |
| BSI46 | 111 | 6.67E+08 | 7.33E+02 | 8.82 | 2.87 | 5.96 | Yes |
| BSI49 | 1018 | 3.00E+08 | 4.67E+04 | 8.48 | 4.67 | 3.81 | Yes |
| BSI51 | 1009 | 3.00E+09 | 1.37E+07 | 9.48 | 7.14 | 2.34 | Yes |
| BSI55 | 709 | 4.67E+08 | 2.67E+06 | 8.67 | 6.43 | 2.24 | Yes |
| BSI56 | 1015 | 1.50E+08 | 4.33E+07 | 8.18 | 7.64 | 0.54 | Yes |
| BSI65 | 699 | 7.67E+07 | 4.33E+07 | 7.88 | 7.64 | 0.25 | No |
| BSI66r | 274 | 2.63E+09 | 1.58E+09 | 9.42 | 9.20 | 0.22 | No |
| BSI66s | 274 | 6.00E+08 | 5.30E+08 | 8.78 | 8.72 | 0.05 | No |
| BSI70 | 395 | 7.00E+08 | 1.10E+07 | 8.85 | 7.04 | 1.80 | Yes |
| BSI72 | 175 | 2.37E+09 | 1.57E+09 | 9.37 | 9.20 | 0.18 | No |
| BSI76 | 244 | 3.67E+07 | 5.67E+06 | 7.56 | 6.75 | 0.81 | Yes |
| BSI82 | 629 | 4.33E+08 | 2.70E+07 | 8.64 | 7.43 | 1.21 | Yes |
| BSI83 | 298 | 5.00E+07 | 1.17E+07 | 7.70 | 7.07 | 0.63 | Yes |
| BSI86 | 267 | 2.67E+08 | 2.33E+08 | 8.43 | 8.37 | 0.06 | No |
| BSI87 | 267 | 5.00E+08 | 1.17E+09 | 8.70 | 9.07 | -0.37 | No |
| BSI93 | 532 | 6.33E+08 | 3.67E+08 | 8.80 | 8.56 | 0.24 | No |
| BSI96 | 395 | 5.67E+08 | 5.00E+08 | 8.75 | 8.70 | 0.05 | No |
| BSI106 | 253 | 4.00E+08 | 4.33E+08 | 8.60 | 8.64 | -0.03 | No |
| BSI109 | 253 | 2.00E+08 | 3.33E+08 | 8.30 | 8.52 | -0.22 | No |
| BSI113 | 897 | 1.23E+09 | 6.67E+08 | 9.09 | 8.82 | 0.27 | No |
| BSI115 | 175 | 8.33E+08 | 8.00E+08 | 8.92 | 8.90 | 0.02 | No |
| BSI123 | 446 | 7.00E+08 | 3.33E+08 | 8.85 | 8.52 | 0.32 | No |
| BSI127 | 369 | 3.67E+08 | 3.67E+08 | 8.56 | 8.56 | 0.00 | No |
| BSI137 | 865 | 1.53E+09 | 1.53E+09 | 9.19 | 9.19 | 0.00 | No |
| BSI146 | 244 | 9.33E+08 | 6.67E+08 | 8.97 | 8.82 | 0.15 | No |
| BSI158 | 508 | 3.00E+07 | 2.67E+06 | 7.48 | 6.43 | 1.05 | Yes |
| BSI159 | 348 | 1.90E+09 | 9.00E+08 | 9.28 | 8.95 | 0.32 | No |
| BSI160 | 348 | 1.20E+09 | 8.33E+08 | 9.08 | 8.92 | 0.16 | No |
| BSI163 | 1017 | 9.00E+08 | 1.10E+09 | 8.95 | 9.04 | -0.09 | No |
| BSI165 | 446 | 5.00E+08 | 7.00E+08 | 8.70 | 8.85 | -0.15 | No |
| BSI168 | 313 | 3.67E+08 | 5.33E+08 | 8.56 | 8.73 | -0.16 | No |
| BSI175 | 1017 | 5.67E+08 | 7.00E+08 | 8.75 | 8.85 | -0.09 | No |
| BSI176 | 532 | 4.67E+08 | 8.67E+08 | 8.67 | 8.94 | -0.27 | No |
| BSI188 | 235 | 4.33E+08 | 5.33E+06 | 8.64 | 6.73 | 1.91 | Yes |
| BSI195 | 235 | 2.33E+08 | 3.67E+08 | 8.37 | 8.56 | -0.20 | No |

## Slide 10
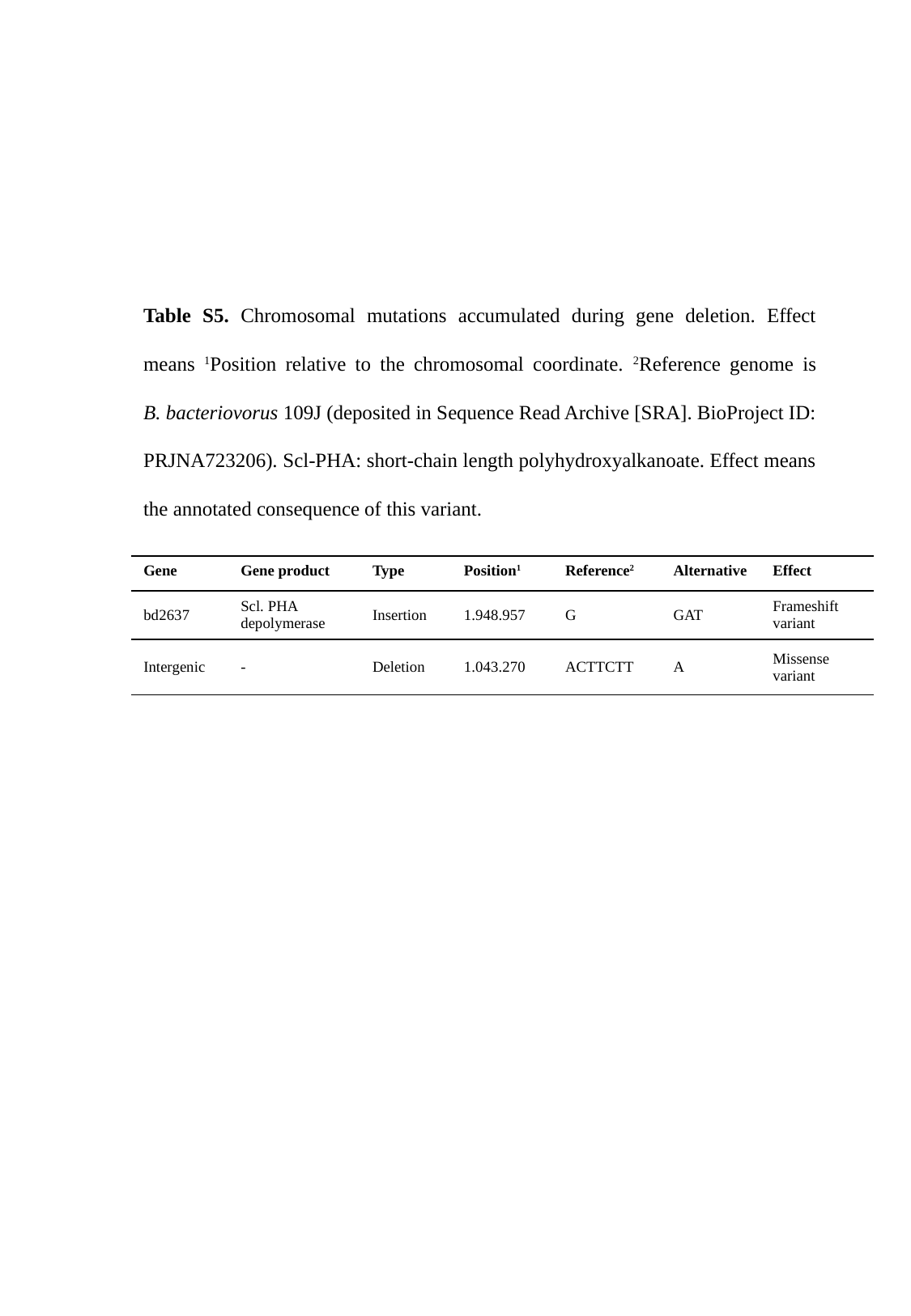

Table S5. Chromosomal mutations accumulated during gene deletion. Effect means 1Position relative to the chromosomal coordinate. 2Reference genome is B. bacteriovorus 109J (deposited in Sequence Read Archive [SRA]. BioProject ID: PRJNA723206). Scl-PHA: short-chain length polyhydroxyalkanoate. Effect means the annotated consequence of this variant.
| Gene | Gene product | Type | Position1 | Reference2 | Alternative | Effect |
| --- | --- | --- | --- | --- | --- | --- |
| bd2637 | Scl. PHA depolymerase | Insertion | 1.948.957 | G | GAT | Frameshift variant |
| Intergenic | - | Deletion | 1.043.270 | ACTTCTT | A | Missense variant |
